# Bending behavior of biomimetic scale covered beam with tunable stiffness scales

**DOI:** 10.1038/s41598-020-74147-0

**Published:** 2020-10-13

**Authors:** Milad Tatari, Soroush Kamrava, Ranajay Ghosh, Hamid Nayeb-Hashemi, Ashkan Vaziri

**Affiliations:** 1 Department of Mechanical and Industrial Engineering, Northeastern University, Boston, MA 02115 USA; 2 Department of Mechanical and Aerospace Engineering, University of Central Florida, Orlando, FL 32816 USA

**Keywords:** Mechanical engineering, Chemical engineering

## Abstract

Biomimetic scales provide a convenient template to tailor the bending stiffness of the underlying slender substrate due to their mutual sliding after engagement. Scale stiffness can therefore directly impact the substrate behavior, opening a potential avenue for substrate stiffness tunability. Here, we have developed a biomimetic beam, which is covered by tunable stiffness scales. Scale tunability is achieved by specially designed plate like scales consisting of layers of low melting point alloy (LMPA) phase change materials fully enclosed inside a soft polymer. These composite scales can transition between stiff and soft states by straddling the temperatures across LMPA melting points thereby drastically altering stiffness. We experimentally analyze the bending behavior of biomimetic beams covered with tunable stiffness scales of two architectures—one with single enclosure of LMPA and one with two enclosures of different melting point LMPAs. These architectures provide a continuous stiffness change of the underlying substrate post engagement, controlled by the operating temperature. We characterize this response using three-point bending experiments at various temperature profiles. Our results demonstrate for the first time, the pronounced and reversible tunability in the bending behavior of biomimetic scale covered beam, which are strongly dependent on the scale material and architecture. Particularly, it is shown that the bending stiffness of the biomimetic scale covered beam can be actively and reversibly tuned by a factor of up to 7. The developed biomimetic beam has applications in soft robotic grippers, smart segmented armors, deployable structures and soft swimming robots.

## Introduction

Scales are among the most apparent and visually striking external geometrically significant features adorning animal integumentary system. Recent investigations have shown that geometry, distribution and stiffness all taken together can result in remarkable enhancement in the substrate response of both naturally occurring fishes^1–8^ as well as bio-inspired synthetic systems^9–13^. These encompass protection against localized loading such as puncture resistance^9,11,14^ as well as modifications to global deformation modes of more slender substrates^15,16^. Thus, scales present an exciting platform for biomimicry. When compared to many other materially engineered solutions, scales directly interface with the work environment and objects, tremendously increasing their impact on performance. Secondly, since source of multifunctionality and extreme mechanical properties are a result of both parent material and topology, it allows for a wide range of properties engineering. One of the primary effect of the biomimetic scales on soft surface is the change in the bending behavior of the overall multi-material structure. Although embedded scales will alter the overall stiffness, the exact relationships between scale properties and distributions are less obvious. Ghosh et al.^15^ provided a contact kinematics model predicting the bending behavior which transitions through linear-nonlinear-rigid regimes (Figure 3b in^15^). The parameters affecting the bending deformation modes are relative stiffnesses of the scales and the substrate, scales overlap ratios, length, contact friction and some other geometrical parameters discussed in previous studies^16–18^. These nonlinear regimes are more universal than pure bending and also reported in non-uniform loads as well as torsion^16,19,20^. Thus clearly, changing the scales properties can lead to a change in the overall behavior of the structure. One of the most convenient parameter of change is the stiffness of the scales. This can change the deformation regimes of the scales themselves, thereby the kinematics. This complex sequence of mechanical effects have not been characterized before. In this paper we study for the first time the effect on bending rigidity, nonlinearity of the bending behavior and the effect of reversible scales deformation on the nonlinear bending behavior of the biomimetic substrate. This material change is introduced using scales with 1 layer of LMPA and 2 layers of LMPAs with different melting points are designed to demonstrate that the concept can be customized for a variety of applications discussed in the manuscript.

Theoretical underpinnings of global deformation nonlinearity have been investigated thoroughly underlying the role of scale size, distribution, orientation and stiffness on soft deformable substrates^6,16–18,21–23^. These models have shown the tailorability of bending properties based on both scale geometry and materials. Such tailorability can be translated to real time tunability by altering material and geometrical characteristics of the scales. However, altering the geometry of fabricated scales is relatively difficult to achieve. In contrast, stiffness control can be achieved through a variety of well-known techniques such as thermally tunable composite wax^24^, vacuum-induced jamming of granular materials^25,26^, electric^27–29^/magnetic^30^ stimuli, smart fluids, magnetorheological (MR) or electrorheological (ER) fluids^31,32^, shape memory materials^33,34^ and rigidity-tuning conductive elastomers^27,29,35^. Encapsulated MR fluids within a structured elastomeric cavity provide stiffness tunability with fast switching time (millisecond range) and relatively high stiffness change ratios ($\sim$ 30×) but also result in high mechanical losses^36^. Shape memory alloys (SMAs) have small stiffness changes (< 4×) and can be directly activated as they are conductive. Nonconductive shape memory polymers (SMPs), can have a high stiffness change ratio (> 100×) and be stimulated using an external heater. The conductive propylene-based elastomer (CPBE) is a conductive elastomer enabling direct joule heating which can be activated in $\sim 6$ s with reversible tensile modulus tunablity of ~ 25× when embedded in a soft elastomer^27,29^. Phase change materials can control stiffness of the scales as they combine extreme stiffness states of smart fluids (soft in liquid phase) and shape memory materials (stiff in solid phase) providing a very large stiffness change ratio^28,37^. On the other hand, the stiffness transition speed is directly linked to the thermal conductivity, and as a result, LMPAs and SMAs are much faster in phase transition than SMPs due to 100× greater conductivity ($18\ \text {W m}^{-1}\ \text {K}^{-1}$ for SMAs and 0.15–0.3 W m$^{-1}$ K$^{-1}$ for SMPs)^34^. Specifically, low melting point alloys (LMPAs) are metals that liquefy at relatively low temperatures (47–62 °C) depending on the alloy composition weight and have elastic moduli in the order of a few GPa. These LMPAs are typically made of bismuth, cadmium, lead, tin and indium with different compositions depending on the desired melting temperature. This class of materials are thus ideal for incorporating into plate like scales to rapidly and reversibly change their stiffness. Scale covered surfaces with dynamically tunable bending stiffness have applications in a wide range of intelligent systems from soft robotics^38^, wearable robotics^39^ to devices with a safe human machine interaction^40^. They provide a new route to realize the next generation of smart man-made segmented armors, puncture/impact resilient and flexible structures^11^, deployable structures, soft robotic grippers^35^, soft biomimetic swimming robots^41^ and any other man-made systems when resilient bending, compliance and robustness are required.

**Figure 1:**
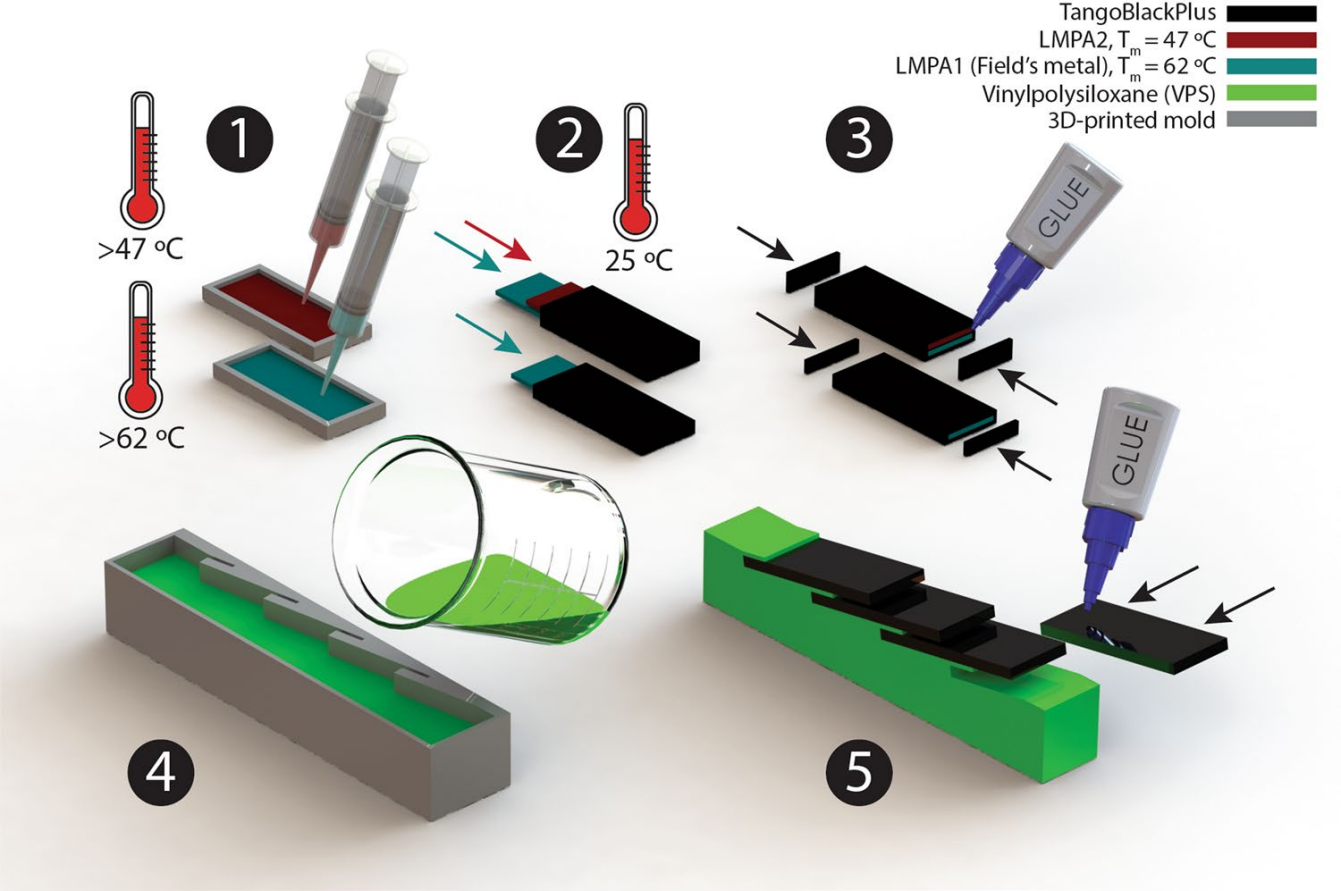
Fabrication process of the biomimetic scale covered beam with tunable stiffness scales. (1) Casting liquid low-melting-point alloys (LMPAs) with melting temperature of 47 °C and 62 °C, respectively, into the soft molds and cooling down to room temperature. (2) Placing the rectangular solid LMPAs into the slots of the 3D-printed soft shell (TangoBlackPlus material) to fabricate two types of scales: double-layer scales (DLS, top) and single-layer scales (SLS, bottom). (3) Bonding two caps to both ends of the scales using Sil-Poxy adhesive to encapsulate both LMPA layers separately in the soft shell. (4) Casting and curing of VPS (Vinylpolysiloxane) elastomer into the 3D-printed mold (Verowhite material) to make the soft substrate. (5) Bonding the fabricated scales to the soft substrate via Sil-Poxy adhesive (the actual fabricated sample has 10 scales).

Here, for the first time, a biomimetic beam with tunable stiffness scales is developed where the stiffness of the scales could be changed via applying heat to the scales with encapsulated LMPAs. The alloy is encapsulated within each plate-like scale in two different morphologies—one with a single layer of LMPA core within the soft TangoBlackPlus polymer shell (single-layer scales or SLS) and another where the interior of the scale is partitioned to contain two cores of the LMPAs (double-layer scales or DLS), Fig. 1. The DLS have two layers of LMPAs that melt at different temperatures providing multistep bending stiffness tunability. After melting is complete, the layers would be essentially fully enclosed chambers containing these liquids, lowering the stiffness of these plate like scales to their lowest permissible values. Bending stiffness of both the SLS and DLS are characterized experimentally, using three-point bending experiments at various scales temperatures. Then, bending behavior of the biomimetic beams with SLS and DLS are investigated at various temperatures. Although, at their extreme phases, the maximum and minimum stiffness of scales translate to similar extremum in bending stiffness of the biomimetic beam, there exists a continuum of stiffness at intermediate temperatures. This is investigated by first heating the samples and then continuously loading them via cyclic three-point bending experiments as they continue to cool down and LMPAs start solidifying (cooling time to room temperature is 20 mins). We find that bending stiffness gains post scales engagement can be tuned by almost an order of magnitude.

## Materials and methods

**Figure 2:**
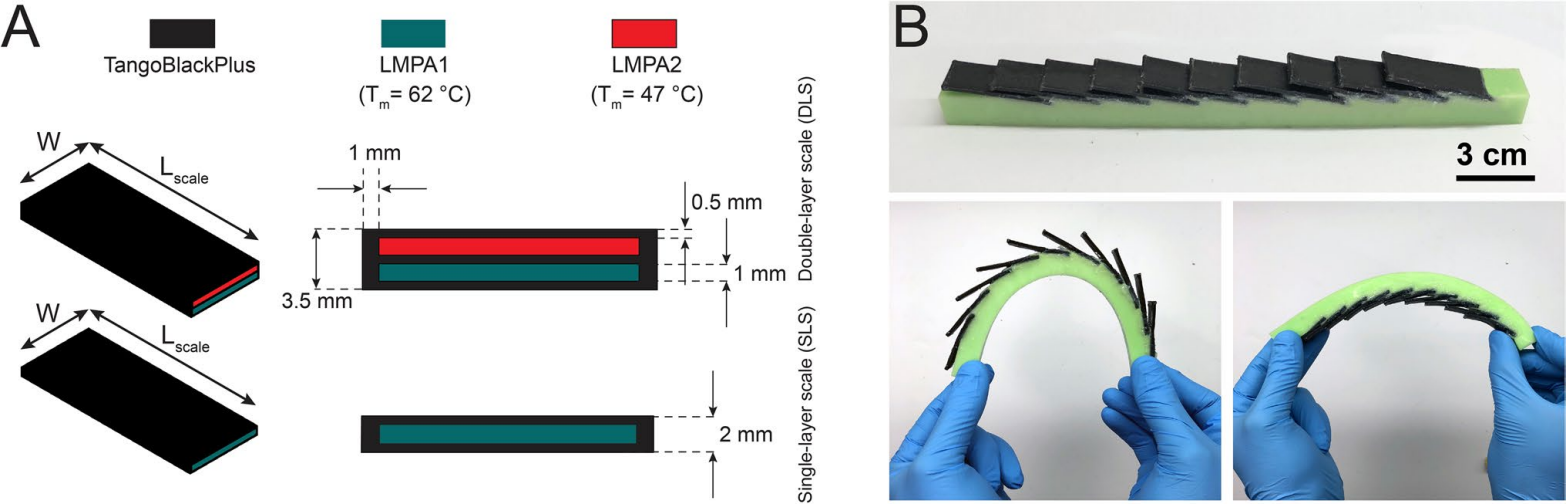
(**A**) Geometrical dimensions of the fabricated SLS and DLS with embedded layers of LMPAs and their dimensions. The width W and length L_scales_ of the scales are 14 mm and 40 mm respectively. (**B**) A tunable biomimetic scale substrate illustrating the contrasting deformation on the scale and plain side.

The biomimetic scale covered beams were fabricated using a multistep manufacturing process (Fig. 1). First, LMPAs (Roto117F/47C and Roto144F/62C, ROTOMETALS Inc. with melting temperature of 47 °C and 62 °C respectively) were cast into the soft molds and cooled down to room temperature. The soft molds for casting liquid LMPAs were printed out of TangoBlackPlus material with a 3D printer (Eden260VS, Stratasys Inc.). In the next step, two stiff rectangular plates (1 mm thickness) are placed into the designed slots of the 3D-printed soft shell (TangoBlackPlus material, Young’s modulus of 0.5 MPa, tensile strength of 1.2 MPa)^42^ to fabricate the two types of scales—SLS and DLS. Then, two caps are glued to both ends of the scales using Sil-Poxy silicon adhesive (Smooth-On Inc.) to encapsulate the LMPA layers in the soft shell. The fabricated scales are illustrated in Fig. 2A. The SLS specimens had only one enclosed core of LMPA1 (Field’s metal, composition by weight: 32.5% bismuth, 16.5% tin, 51% indium; T_m_ = 62 °C). The two cores of DLS had one filled with LMPA1 and another of LMPA2 (composition by weight: 44.7% bismuth, 5.3% cadmium, 22.6% lead, 8.3% tin, 19.1% indium; T_m_ = 47 °C). When scales are at room temperature, all LMPA layers are solid and scales are stiff. When the scales are heated up to the range of $47 \; ^\circ \text {C}< \text {T}_{\text{scales}} < 62 \; ^\circ \text {C}$ only LMPA2 melts while LMPA1 remains solid. As the temperature increases beyond 62 °C, both of these scales would be in their liquid state. Therefore, the stiffness of SLS specimens straddles between stiff and soft states whereas for the DLS specimens, three significant states exist—stiff, partially soft (only one chamber molten) and fully soft (both chamber molten). Note that in the fully solid (stiff state), the DLS will be inherently stiffer than the SLS due to higher volume fraction of metal as well as greater thickness of the scale. The dimensions of scales fabricated for this work are 40 mm length, 14 mm width for both types of scales and a thickness of 2 mm and 3.5 mm for SLS and DLS respectively.

To fabricate the soft substrate, a 3D-printed mold (Verowhite material) is filled with VPS elastomer (Vinylpolysiloxane, elite double 32, Zhermack Inc.) and then cured in room temperature for 20 mins. VPS elastomer is prepared with a 1:1 weight ratio of base elastomer to catalyst agent and mixed for 1 min. This results in a soft substrate with channels defined by the features to fit the fabricated scales. In the final step, scales are bonded to the substrate using Sil-Poxy adhesive. Both SLS and DLS are fixed at an angle of 10° and embedded into the substrate by 6 mm.

Figure 2B illustrates a fabricated biomimetic beam with 10 SLS, embedded with Field’s metal (T_m_ = 62 °C, Young’s modulus of 9.25 GPa)^43^ and manual illustration of bending deformation on scale side (side with scales) and plain side (side without scales). Clearly scales engagement on the scale side restricts curvature when compared with the plain side (see Supplementary Figure S1 for an illustration of bending deflection of the biomimetic beam as a cantilever). To characterize bending stiffness of the specimens, three-point bending experiments are performed using an Instron 5943 with a 1 kN load cell under displacement control at crosshead speed of 0.05 mm/s for the scales only and 0.5 mm/s for the biomimetic beam. The temperature changes is brought about by placing the biomimetic beam on a corning hot plate (Model PC-400D) and setting the hot plate temperature according to the experiments (activation time is 2 mins). Throughout the experiments, scales temperature is monitored using a thermal camera (FLIR ONE Pro, FLIR Inc.) (see Supplementary Figure S2). Next, in addition to discrete stiff, partially soft and soft states, we also aim to investigate the continuous bending stiffness tunability. This can be achieved by first heating the scale to achieve the extreme soft state and then letting it cool down and thus transition back to the more solid (stiffer) states and characterizing its load displacement characteristic. This motivates a cyclical loading scheme where the preheated specimens are subjected to repeated loads as it cools down. The cyclical experiments are conducted using Instron 5943 with a 1 kN load cell, a crosshead speed of 2 mm/s with a maximum deflection of 15 mm for all cases. There is no dwell time for this loading at any stage of loading.

## Results and discussion

**Figure 3:**
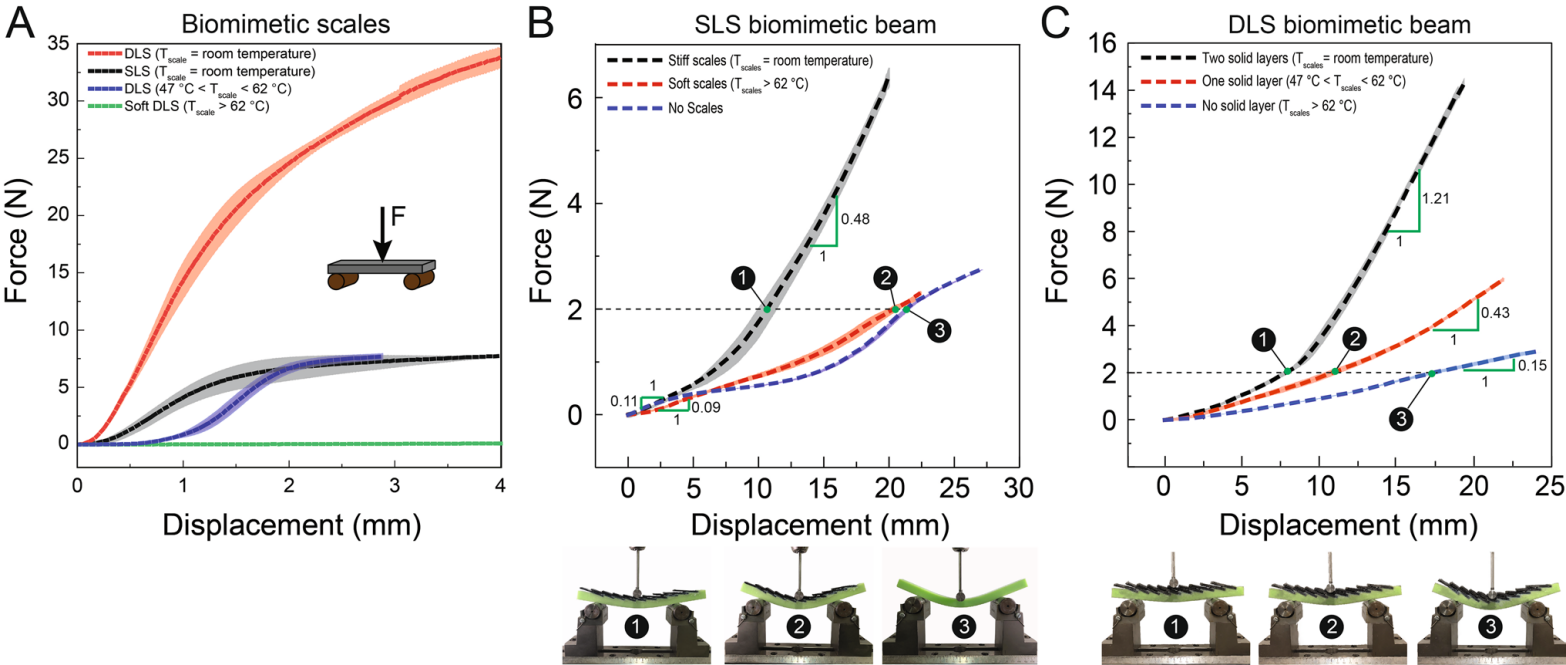
(**A**) Comparative force-displacement plots for three-point bending experiments conducted on the fabricated scales for DLS and SLS and soft scales to show their bending stiffness behavior. DLS can provide stiffness tunability depending on the phase of the embedded LMPA layers (liquid or solid). When both layers of DLS are in solid phase, the stiffness gain is maximum (red line). When the top layer is melted, the stiffness is drastically reduced, following approximately the same trend as SLS (blue and black lines which are only shifted in x axis reflecting later engagement for DLS). Force-displacement plots for a three-point bending experiment conducted on the fabricated sample with (**B**) SLS (**C**) DLS. (**B**) Rigid scales are engaged when the scale covered beam bends and after scale engagement, the system stiffness increases to 0.48 N/mm, however, soft scales can conform to soft substrate such that scale covered beam stiffness is in the order of that of pure soft substrate with no scales. (**C**) DLS provide multistep bending stiffness tunablity of the biomimetic beam depending on the rigidity of the scales. When the scales are completely stiff it has the highest stiffness, and as the scales heated up to between 47 and 62 °C, scale covered beam stiffness drops since the top layer will be liquid. Lowest stiffness of the system is achieved when both LMPA layers of scales are in the liquid phase. Insets: configurations of scale covered beam at 2 N force for different states of scales stiffness.

We now investigate the deformation behavior of the fabricated samples. First, we characterize the tunable bending rigidity of the individual SLS and DLS using a three-point bending test, Fig. 3A. This figure shows that in the stiff state (low temperature) DLS (red line) are stiffer than SLS (black line) whereas the fully soft DLS scales where all the LMPAs have melted (green line) show no bending stiffness as expected. As the temperature of the stiff DLS is increased, it enters the partially soft state leading to a dramatic reduction in bending stiffness (blue line). Once the DLS are heated up to $47 \; ^\circ \text {C}< \text {T}_{\text{scales}} < 62 \; ^\circ \text {C}$, LMPA2 melts and its initial stiffness, reduces significantly. This has shifted its force-displacement plot to the right by an amount of 1 mm (thickness of layer 2) since the top layer is LMPA2 which now has been melted. Besides this horizontal shift in force-displacement plot, the trend is similar to that of SLS (black and blue lines). For instance, at 2.80 mm displacement, force value is 7.627 ± 0.256 N and 7.20 ± 0.65 N for DLS (liquid layer 2) and SLS respectively. It should be noted that the bending stiffness behavior of the scales is completely reversible since as scales are cooled down to room temperature, they recover their stiffness.

Next, we characterize the bending behavior of scale covered beams with these embedded scales. This is illustrated in Fig. 3B (SLS biomimetic beams) and Fig. 3C (DLS biomimetic beams) and obtained from three-point bending experiments. Here, bending stiffness is defined as the slope of force-displacement plots. For SLS biomimetic beams, Fig. 3B, beam with stiff scales has a low stiffness 0.11 N/mm before the scale engagement commences. Once the scales are engaged, the stiffness sharply increases to 0.48 N/mm (black line). On the contrary, when the scales are heated to trigger the softer state, the bending stiffness is lower as expected (red line). These can be contrasted with the bending behavior of the plain beam (blue line). In the soft state, even the initial stiffness of the biomimetic beam is slightly lower than the plain beam. This is because the loss of stiffness lowers the effective stiffness of the top embedding layer of the biomimetic beam. As the bending load increases, the stiffness gap between the softer beam and the plain beam narrows down as the liquid at the top layer is compressed more, leading to higher internal pressures at greater bending loads. This is reflected in the convergence of the red and blue lines in the figure at higher loads. Interestingly, whereas the stiff scales system continues to show increase in the stiffness, the softer system plateaus, with little nonlinear gains at higher loads. Insets show bending configurations of scale covered beam under a force of 2 N for different states of stiff, soft and no scales.

Characterization of DLS biomimetic beam under bending is illustrated in Fig. 3C. In this case, like the previous case of SLS biomimetic beam, the stiff state (black line) substantially differs from the plain beam (blue line) indicating gains from scales engagement. The gains in stiffness from engagement at a given displacement is almost double (1.21 N/mm) that of SLS beam (0.48 N/mm), Fig. 3B. This increased stiffness is partly a reflection of higher stiffness of the DLS themselves as well as the higher substrate resistance to scale movement due to greater thickness of the double layer scales^15^. When temperature is increased, the LMPA2 melts, transitioning the system into a partially soft state, reducing the stiffness substantially (red line). Since one of the chambers continues to be solid, the overall bending stiffness is intermediate between a beam with soft scales and fully stiff beam. As the temperature increases beyond 62 °C, both LMPA chambers completely liquefy resulting in the lowest bending stiffness value of the DLS biomimetic beam exhibiting stiffness of 0.15 N/mm.

**Figure 4:**
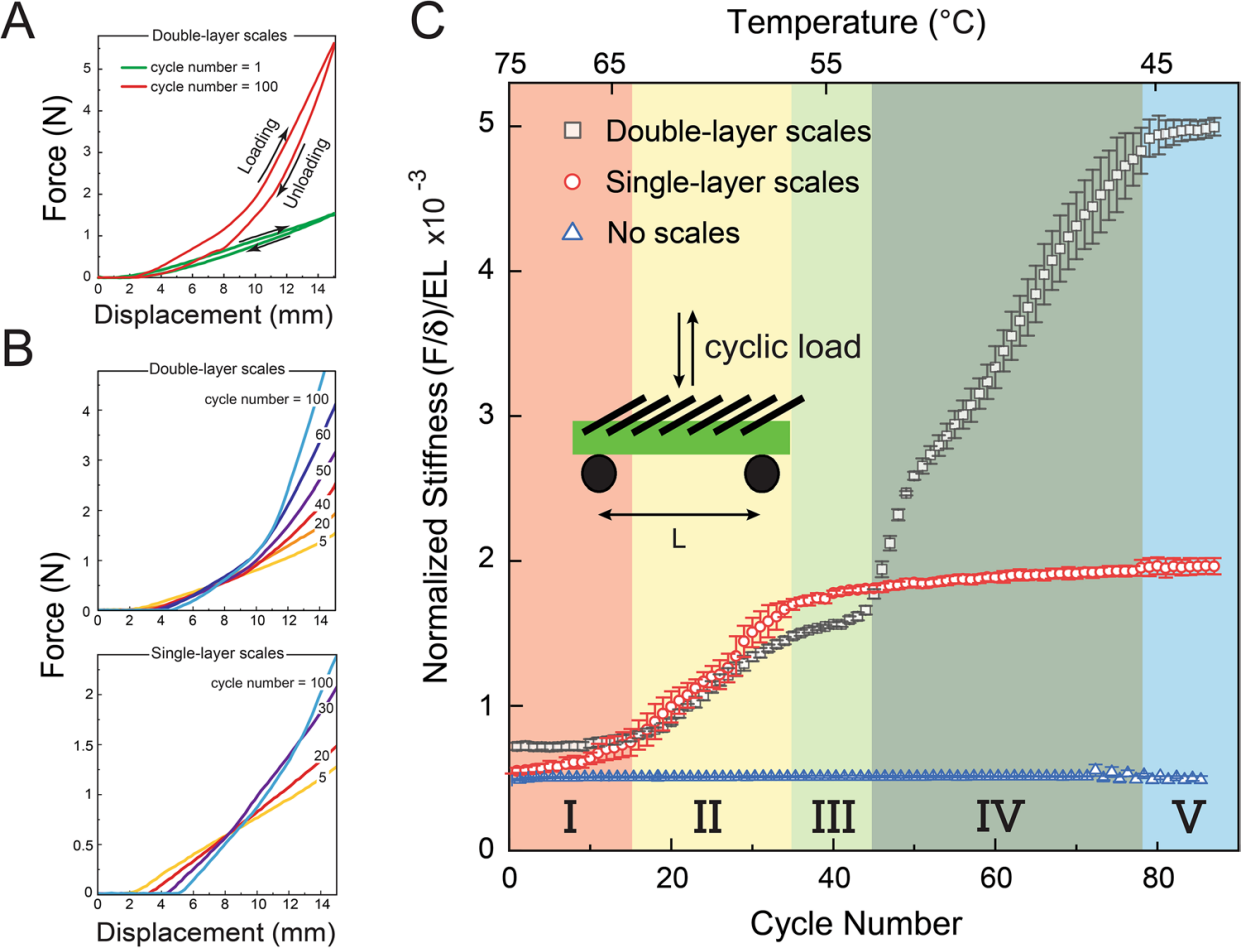
(**A**) Force-displacement plot of three-point bending experiments: loading and unloading curves of cycle numbers = 1 (soft scales) and 100 (rigid scales). Experiments are conducted on a scale covered beam with double-layer scales (DLS). (**B**) Cyclic force-displacement plots of the tunable stiffness SLS and DLS biomimetic beams. In the first cycle, scales temperature is 75 °C (soft scales) and decreases as the experiment continues (experiments are conducted at room temperature). A limited number of cycles have been selected to show force-displacement plots as the cycle number increases for SLS (bottom) and DLS (top). Once the scales are stiff, force-displacement plots of the system do not change as cycle number increases. (**C**) Normalized stiffness of the SLS and DLS biomimetic beams after scale engagement is plotted versus cycle number. The stiffness is normalized by dividing with Es × L value. As cycle number increases, scales temperature decreases and for the SLS beam after 37 cycles, scales will be rigid and system stiffness does not change in the next cycles. For DLS, the biomimetic beam stiffness is constant in regions III and V . Between cycle number of 37 and 43, layer 1 is rigid ($47 \;^\circ \text {C} < T_{\text{scales}} < 62 \;^\circ \text {C}$) and then as it continues, layer 2 starts solidifying such that after 78 cycles it will also be solid and scale covered beam stiffness will be constant. Bending stiffness of the DLS beam can be tuned up to 7× depending on the scales stiffness.

In addition to these temperature triggered states, a wider spectrum of stiffness response exists which can be exploited for a more continuous tunability. Figure 4B investigates continuously changing bending behavior as the scales transition continuously from a softer (hot) to a stiffer (cold) state. This figure shows cyclic three-point bending experiments conducted on both DLS and SLS biomimetic beams starting at T_scales_ = 75 °C. We note that in cyclic three-point bending experiments for both soft and stiff states, a hysteresis between loading and unloading curves is observed which is due to the friction at two supports and formation of indented deformation of the scales as they transition from liquid to solid^44^ (Fig. 4A). The main focus of the current work is on the loading cycle of three-point bending experiments to investigate the effect of scale engagement. As the cycle number increases, scales temperature decreases due to thermal loss to environment (experiments are conducted at room temperature) and different layers of LMPA (layer 1 and 2) start solidifying. This is evident in the figure where cycle numbers for DLS and SLS biomimetic beams are shown for comparison. For DLS biomimetic beams (Fig. 4B), top), first, layer 1 solidifies because of higher melting temperature (T_m_ = 62 °C), then the layer 2 phase (T_m_ = 47 °C) changes to solid and after 78 cycles, complete solidification occurs and therefore bending behavior of the biomimetic beam did not change anymore. For SLS (T_m_ = 62 °C), complete stiffness was achieved much earlier, at 37 cycles, after which there was no change in bending stiffness of the biomimetic beam (Fig. 4B, bottom). The relatively straight load displacement plots (indicating roughly constant stiffness) of this figure allows us to unify this continuous change in bending stiffness using a phase diagram, which plots normalized bending stiffness with cycle number, Fig. 4C. The bending stiffness is the slope of the force-displacement plots after scale engagement. The stiffness is normalized by dividing with Es × L where Es = 1.4 MPa is the elastic modulus of the substrate^15^ and L = 140 mm is the distance between two support pins in the three-point bending experiments. In this plot five distinct regions of stiffness emerge. In region I, scales are completely soft and DLS biomimetic beam has the highest stiffness and the pure substrate, which has no scales has the lowest stiffness, with the SLS biomimetic beam in between. Note that, the bending stiffness of a scale covered beam with no scales does not change with temperature and is constant (blue points). Region II shows a large increase in bending stiffness of both SLS and DLS biomimetic beams, due to the onset of liquid-solid transition of LMPA1 of the scales. This phase is completed with the LMPA1 completely solidifying. This is the stiffer limit of SLS and occurs after 37 cycles. After this region, the stiffness of either SLS or DLS biomimetic beams do not change as no immediate phase change occurs. Thus region III, sets the final limit on the stiffness of the SLS biomimetic beam, indicating long term stiffness stabilization for this type of specimen. This region ends, when LMPA2 begins to solidify with falling temperature, further raising the stiffness of the second layer of the DLS. This is the onset of region IV marked by a rapid stiffness increase of the DLS. The stiffness continues to increase with each cycle as temperature continues to fall until this chamber is completely solidified and the DLS biomimetic beam attains its maximum stiffness for a given deformation. This complete solidification occurs after about 78 cycles and is characterized by complete long-term stabilization of the beam stiffness for DLS designs. In addition, the proposed biomimetic scale covered beam has been shown to exhibit consistent mechanical behavior after more than 40 activation cycles and cooling down over a span of three months, which shows the robustness of the biomimetic beam. Although accelarated cooling has not been investigated as it is beyond the scope of the paper, in practice, cooling accelaration is possible by a number of design features such as using an air gun, water spray or substrate embedded cooling mechanisms.

## Conclusion

In this paper, we have investigated for the first time, a biomimetic beam covered with tunable stiffness scales to achieve a continuous stiffness change of the underlying substrate. Scales stiffness is tuned by enclosing phase change LMPA materials into a 3D-printed soft shell. Such materials were able to reversibly transition between the extreme stiffness states through liquefying and solidifying with temperature, thereby also altering the scale stiffness. We exploited this feature to endow a large range of tunability to the fabricated scales by enclosing them into a single (SLS) and double layer (DLS) architecture. The SLS have 1 layer of phase change alloy, while DLS have two layers of phase change materials with different melting temperatures to provide multistep stiffness tunablity. These scales were then affixed on one side of a beam like soft polymeric substrate, resulting in a biomimetic scale covered beam of two different types. Bending stiffness tunability of these scale covered beams are then experimentally characterized using three-point bending tests under thermal loads to activate the phase change behavior. We find that there is a continuum of bending stiffness values (after scale engagement) of the biomimetic beam between extreme states of embedded LMPA layers (solid or liquid). It is also shown that the stiffness of the tunable stiffness biomimetic beam can be dynamically and reversibly tuned as high as 7× as phase change of LMPA layers from liquid to solid. Scales embedding itself will lead to increase in stiffness. This effect is however, distinct and already investigated in some detail in literature^15^, including a comparison with the scales engagement (sliding effect)^12,19^. The effect of pure embedding is primarily a composite effect and measurable even before engagement. The effects of engagement are clearly visible by sharp increases in stiffness which is not the case with only inclusions^12,19^.

It may be possible to fabricate the tunable stiffness scales of scale covered systems using alternative ways by making embedded channels of LMPAs enabling direct joule heating of individual scales. It provides localized stiffness tuning of biomimetic surfaces to dissipate local impact forces with optimized energy consumption that have applications in development of robust structures. Moreover, in the current study, SLS and DLS biomimetic beams are developed whereas it can have multi-layer of tunable stiffness materials to achieve the desired scales stiffness depending on the application. In the future, an aspiring goal would be to apply the developed biomimetic beam in a “soft robotic gripper” or “smart man-made segmented armors” to fully integrate sensing, actuations and control strategies to mimic the adaptability of biological systems. As an example, a biomimetic beam coupled with soft pneumatic actuators^45^ can be potentially employed to tune the distance between scales (controlling scales overlap ratio) and develop adaptive morphing robotic grippers.

## Supplementary information

Supplementary Information 1.

Supplementary Information 2.
