# Supplementary material for: Bending behavior of biomimetic scale covered beam with tunable stiffness scales
Source: Sci Rep. 2020 Oct 13;10:17083. doi: 10.1038/s41598-020-74147-0 (PMC7554036; doi:10.1038/s41598-020-74147-0)
Supplement: Supplementary file 1 — Supplementary Information 1. [file 41598_2020_74147_MOESM1_ESM.pdf]

Supplementary Information

Bending Behavior of Biomimetic Scale Covered Beam with Tunable Stiffness Scales

Milad Tatari[1], Soroush Kamrava[1], Ranajay Ghosh[2], Hamid Nayeb-Hashemi[1], and Ashkan Vaziri[1,*]

[1]Department of Mechanical and Industrial Engineering, Northeastern University, Boston, MA 02115, USA.

[2]Department of Mechanical and Aerospace Engineering, University of Central Florida, Orlando, FL 32816, USA.

*avaziri2@gmail.com

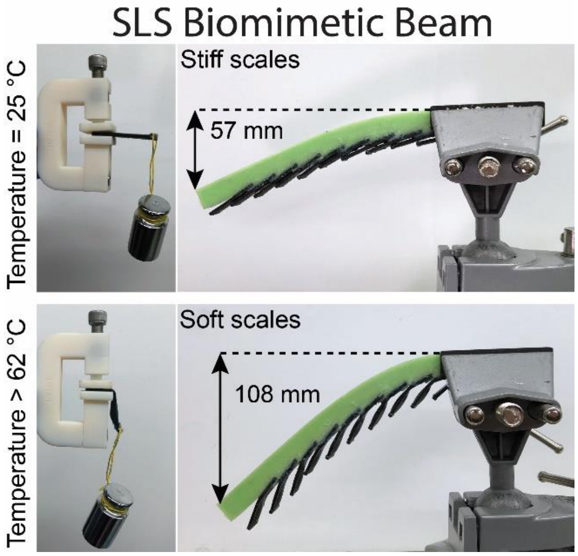

Figure S1. Deflection of the developed SLS biomimetic beam as a cantilever due to its weight, stiff scales (top), soft scales (bottom).

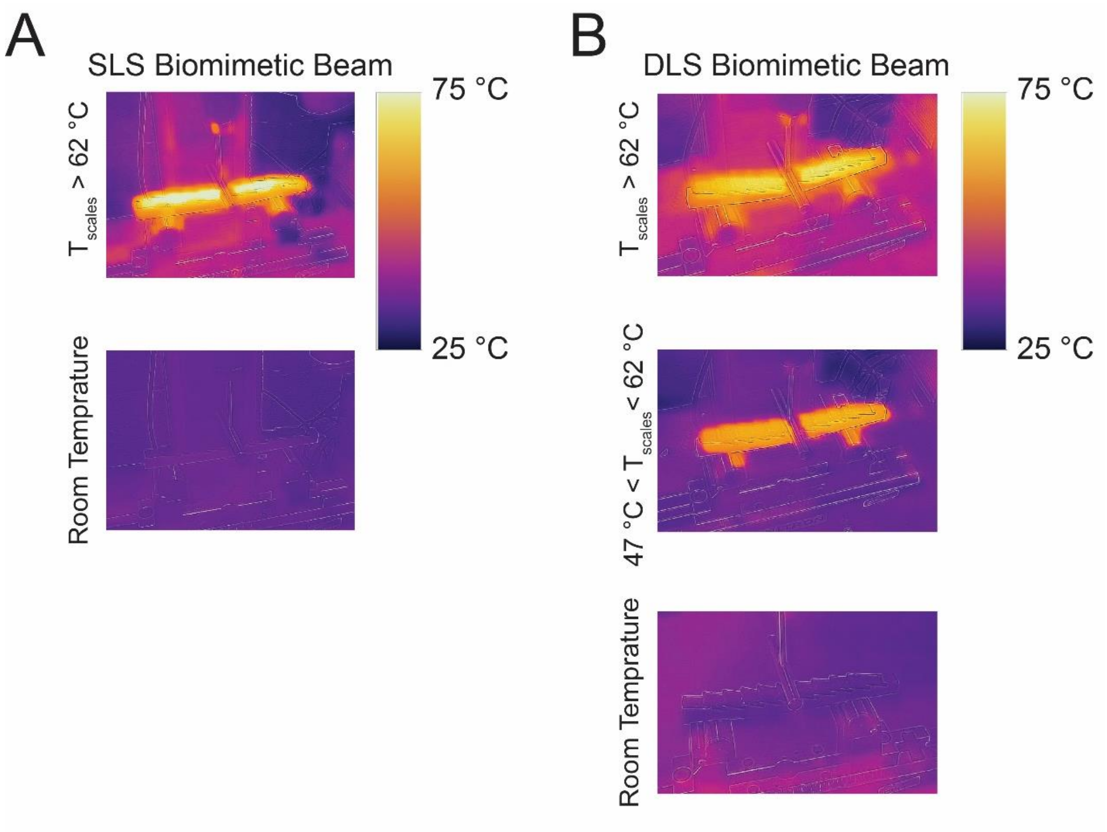

Figure S2. Scales temperature distribution captured by the thermal camera: (A) SLS biomimetic beams and (B) DLS biomimetic beam.
